# MDV-like endogenous viral elements act as immune rheostats in *Aedes* cells by modulating defensin A-mediated responses to arboviruses

**DOI:** 10.1128/msphere.00520-26

**Published:** 2026-09-11

**Authors:** Jie Xiao, Tiantian Wei, Hongzheng Lu, Chunyuan Li, Mei Chen, Peiwen Liu, Xiao-Guang Chen, Zetian Lai, Ruiwen Ren, Jinbao Gu

**Affiliations:** 1 School of Public Health, Southern Medical University 569910, Guangzhou, Guangdong, China; 2 Guangdong Provincial Key Laboratory of Tropical Disease Research, Southern Medical University 658728, Guangzhou, Guangdong, China; 3 Key Laboratory of Infectious Diseases Research in South China (Southern Medical University), Ministry of Education, Southern Medical University 70570, Guangzhou, Guangdong, China; 4 Laboratory Medicine Centre, Shenzhen Nanshan People's Hospital 194030, Shenzhen, People’s Republic of China; 5 Shenzhen University Medical School 481870, Shenzhen, People’s Republic of China; 6 Center for Disease Control and Prevention of Southern Theater Command, Guangzhou, China; 7 Guangzhou Center for Disease Control and Prevention 214164, Guangzhou, China; University of Southern Mississippi, Hattiesburg, Mississippi, USA

**Keywords:** *Aedes aegypti*, Aag2 cell, mosquito densoviruses, endogenous viral elements, defensin A

## Abstract

Mosquito cell lines are essential tools for arbovirus research. Endogenous viral elements (EVEs) are prevalent in mosquito genomes, yet their functional effects on host immune responses remain unclear, potentially complicating experimental interpretations. In this study, we systematically characterized endogenous mosquito densovirus-like elements (EMLs) within the *Aedes aegypti* Aag2 cell line and found that these endogenous EMLs are transcriptionally active but translationally defective. The silencing of EML transcripts significantly diminished the replication of Zika virus (ZIKV), Japanese encephalitis virus (JEV), and chikungunya virus (CHIKV), while transiently increasing dengue virus 2 (DENV-2), thereby indicating a virus-dependent regulatory mechanism. Mechanistically, RNA sequencing after EML interference, alongside plasmid-based mimic expression, demonstrated that EML transcripts downregulate defensin A, an antimicrobial peptide produced by mosquitoes. Functional assays using synthetic defensin A showed that this peptide differentially regulates arboviral infection. Binding assays and structural modeling further supported its interaction with viral envelope proteins, while stage-restricted infection assays revealed distinct stages of action: defensin A enhanced adsorption of ZIKV, JEV, and CHIKV, but did not promote DENV-2 adsorption or entry, and instead reduced DENV-2 RNA accumulation at the post-entry replication stage. Our findings highlight a previously unrecognized role of densovirus-derived EVEs in mosquito innate immunity, extending their functional scope from the well-established PIWI-interacting RNA-mediated antiviral defense to the regulation of antimicrobial peptide-associated immune pathways. These findings emphasize the necessity of accounting for EVE activity when analyzing data derived from mosquito cell lines, and suggest that related EVE-mediated immune regulation may contribute to arbovirus dynamics in mosquitoes.

**IMPORTANCE:**

Mosquito-borne viruses such as dengue, Zika, Japanese encephalitis, and chikungunya continue to threaten human health worldwide. Laboratory studies often use *Aedes aegypti* cell lines to investigate how these viruses interact with their mosquito hosts. Here, we show that the genomes of these cells contain endogenous viral elements derived from mosquito densoviruses. Far from being inert fossils, these sequences are transcriptionally active and regulate mosquito immunity by suppressing the antimicrobial peptide defensin A. This immune modulation influences the replication of different arboviruses in opposite ways, enhancing some while restricting others. Our findings reveal that integrated viral elements can shape the outcome of arbovirus infection, with important implications for interpreting mosquito cell culture experiments and for evaluating endogenous viral element-mediated immune regulation in mosquito–virus interactions.

## INTRODUCTION

Mosquito-borne viruses (MBVs), including dengue virus (DENV), Zika virus (ZIKV), and chikungunya virus (CHIKV), remain a major global health threat, driven by globalization and the spread of competent mosquito vectors. Mosquito cell culture systems provide essential platforms to dissect vector–virus dynamics, define host determinants of infection and immunity, and assess interactions among co-infecting pathogens within the vector. The first continuous mosquito cell line was established from *Aedes aegypti* larvae in the 1960s (1). Since then, multiple lines have been developed, among which the *Ae. albopictus*-derived C6/36 and the *Ae. aegypti*-derived Aag2 lines are most widely used. C6/36 cells, originating from larval tissue of *Ae. albopictus*, have proven highly permissive for arbovirus isolation and amplification (2, 3), while Aag2 cells, derived from *Ae. aegypti* embryos, are widely employed for studying mosquito immunity and host–virus interactions (4). Both lines have been fully sequenced, providing comprehensive genomic resources for vector biology research (5, 6).

Despite their utility, mosquito cell lines present important challenges. Persistent infections with insect-specific viruses (ISVs) are now recognized as widespread in laboratory-maintained lines. For example, Aag2 cells harbor cell-fusing agent virus, Phasi Charoen-like virus (7), and Aedes anphevirus (6), while other lines contain diverse ISVs such as Culex Y virus, Flock House virus, and additional bunyaviruses and flaviviruses (7, 8). These covert infections can interact with invading arboviruses, and co-infections have been shown to significantly alter arbovirus replication, complicating interpretation of experimental results. In addition to active infections, viral sequences can integrate into host genomes, giving rise to endogenous viral elements (EVEs). Mosquito genomes harbor abundant EVEs derived from both ISVs and arboviruses (9). Far from being inert relics, many EVEs are transcriptionally active and generate PIWI-interacting RNAs (piRNAs), suggesting a role in EVE-derived RNA interference immunity (10–12). This dual presence of persistent ISVs and EVEs highlights that mosquito cell lines are not neutral systems but dynamic players in antiviral defense and vector–virus coevolution.

Mosquito densoviruses (MDVs) are small, non-enveloped, single-stranded DNA viruses of the family Parvoviridae and exemplify this complexity. MDVs are pathogenic to mosquitoes, frequently detected in cell lines, laboratory colonies, and wild populations (13, 14). Given their ubiquity, interactions between MDVs and arboviruses in mosquito systems are inevitable, and several studies have demonstrated that such interactions can profoundly influence arbovirus replication *in vitro* and *in vivo*.

In our previous analysis of Aag2 cells, we identified nearly full-length MDV-derived endogenous viral elements integrated into the Aag2 genome; hereafter, these sequences are referred to as endogenous MDV-like sequences (EMLs). Building on this observation, the present study investigates the genomic organization, copy number, and transcriptional potential of MDV-like elements, and examines their impact on arbovirus replication. By elucidating how these endogenous viral elements interface with mosquito innate immunity, we aim to provide new insights into the hidden variables that shape arbovirus research outcomes in cell lines and potentially influence virus dynamics in natural mosquito populations.

## RESULTS

### EMLs are integrated within the Aag2 genome

**Fig 1:**
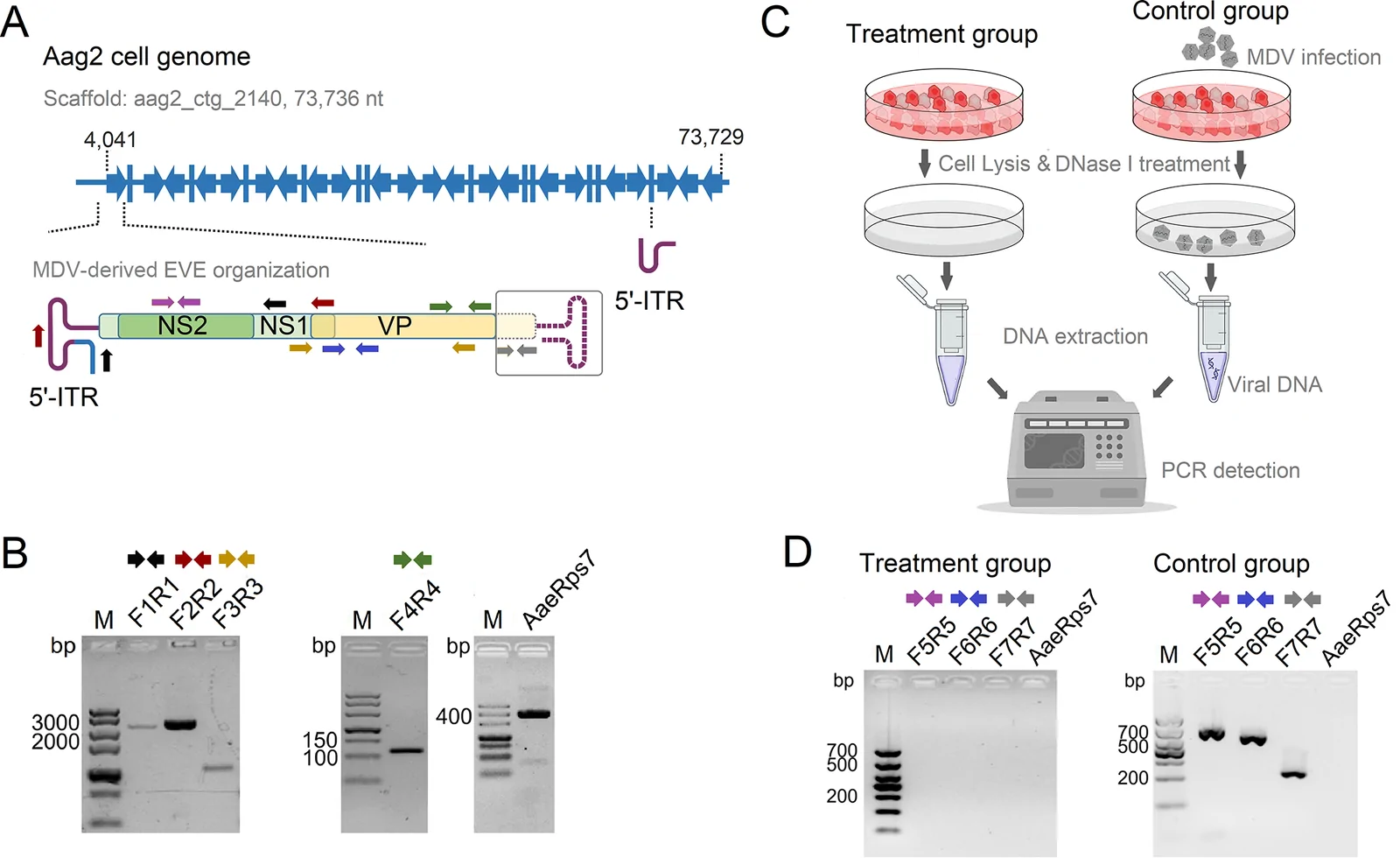
Identification and validation of EMLs in the Aag2 genome. (**A**) Schematic representation of MDV-derived EML organization in scaffold aag2_ctg_2140 (73,736 nt). Integration spans positions 4041–73729, containing multiple tandem EMLs. Many loci include nearly full-length viral sequences, comprising the 5′ ITR, NS2 and NS1 genes, and a partial VP gene, while lacking the 3′ ITR. Primer positions (arrows) used for PCR validation are indicated. (**B**) Agarose gel electrophoresis of PCR products amplified from Aag2 genomic DNA using four primer sets (F1R1–F4R4). Amplicon sizes matched predictions, confirming EML integration. *AaeRps7* was amplified as an endogenous control. (**C**) Experimental workflow. Aag2 genomic DNA (treatment group) was subjected to DNase digestion to eliminate non-encapsidated DNA, while AaeDV-infected Aag2 cells (control group) served as a source of encapsidated viral DNA. (**D**) PCR detection of MDV sequences. No amplification was observed in DNase-treated Aag2 genomic DNA (treatment), whereas viral DNA was readily detected in infected controls. *AaeRps7* was used as an internal control.

To determine whether EMLs are present in the *Aedes aegypti* Aag2 genome, AaeDV sequences were queried against the Aag2 assembly using BLASTn. A total of 34 significant hits (*E*-value < 1 × 10^−6^) were identified in scaffold aag2_ctg_2140 (73,736 nt; Table S3). Because this scaffold was omitted from the updated Aag2 genome assembly, its complete sequence is provided in Table S1C. These EMLs were organized in tandem, spanning nucleotides 4,041–73,729 of the scaffold. Notably, 22 loci encompassed nearly the full-length viral genome, including the 5′ inverted terminal repeat (ITR), the entire nonstructural (NS) protein gene region, and a partial viral particle (VP) gene cassette, lacking only the 3′ ITR. An additional 12 loci corresponded to truncated 5′ ITR fragments, hereafter referred to as endogenous ITRs (EITRs) (Fig. 1A). To experimentally validate these integrations, we designed four primer sets targeting the predicted EML regions (Fig. 1A). PCR amplification from Aag2 genomic DNA yielded products of the expected sizes (Fig. 1B), and Sanger sequencing confirmed that the amplicons were consistent with the genomic assembly (data not shown).

Because mosquito cell lines are known to harbor adventitious viral infections (15), we further excluded the possibility that these signals arose from contaminating viral DNA. To this end, Aag2 genomic DNA was subjected to DNase digestion to remove non-encapsidated DNA, followed by PCR detection. As a negative control, we tested Aag2 DNA, while an AaeDV-infected Aag2 culture served as a positive control. As shown in Fig. 1C, PCR signals corresponding to EMLs were absent in DNase-treated Aag2 DNA, while encapsidated viral DNA was readily detected in the infected control (Fig. 1D). Collectively, these findings demonstrate that MDV-derived EMLs are stably integrated within the *Ae. aegypti* Aag2 cell line genome.

### Characteristics of EMLs in the Aag2 genome

**Fig 2:**
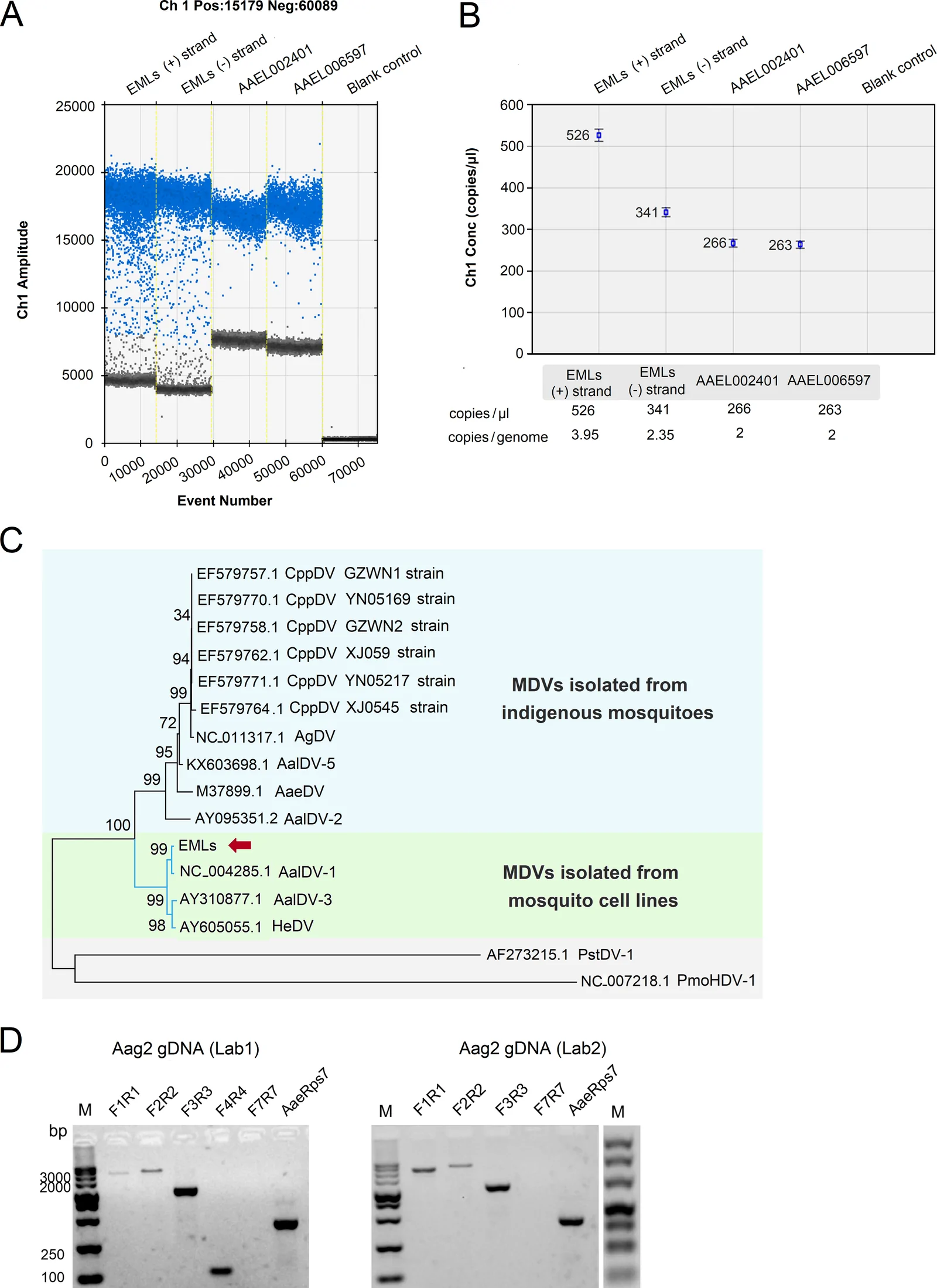
Copy number determination and phylogenetic placement of MDV-derived EMLs in Aag2 cells. (**A**) One-dimensional ddPCR plots showing fluorescence amplitude of droplets for EMLs mapped to the (+) strand, (−) strand, and single-copy control genes (*AAEL002401* and *AAEL006597*). QuantaSoft software classified positive droplets (blue) and negative droplets (black). (**B**) Copy number quantification of EMLs and control genes. Bars indicate mean concentrations (copies/μL), with error bars representing the maximum and minimum Poisson distribution at the 95% confidence interval generated by QuantaSoft. Estimated genomic copies per diploid genome are shown below each bar. (**C**) Phylogenetic analysis of NS1 protein sequences places the Aag2 EMLs (red arrow) as close relatives of AalDV-1, previously isolated from *Ae. albopictus* C6/36 cells. MDVs from cell lines clustered separately from those isolated from indigenous mosquitoes. The tree was rooted with PmoHDV-1 and PstDV-1. Bootstrap values (>70) are indicated. (**D**) PCR validation of EML integration in Aag2 genomic DNA from two independent laboratories. Consistent amplification with multiple primer sets (F1R1–F7R7) confirmed the stable presence of EMLs in Aag2 cells. *AaeRps7* served as an internal control.

Given that 22 nearly full-length EMLs were organized in tandem within aag2_ctg_2140, we next sought to exclude the possibility that these repeats were artifacts of genome assembly. To this end, we used droplet digital PCR (ddPCR) to quantify the copy number of MDV-derived sequences, including loci mapped to the plus (+) and minus (−) strands of the reference genome (excluding EITRs). Two single-copy genes, *AAEL002401* and *AAEL006597*, served as internal controls (present in two copies per diploid genome). ddPCR analysis revealed that the Aag2 genome harbors ~3.95 copies of EML sequences on the (+) strand and ~2.35 copies on the (−) strand, corresponding to an estimated three genomic copies of EMLs per diploid genome [two from the (+) strand and one from the (−) strand] (Fig. 2A and B).

To assess the evolutionary relationship of these EMLs, we performed phylogenetic analysis based on the conserved NS1 protein. The EMLs clustered closely with AalDV-1 (synonym: Aedes albopictus parvovirus, AaPV), originally isolated from *Ae. albopictus* C6/36 cells in 1992 (16). The phylogeny revealed two distinct MDV clades (17): one consisting of AalDV-1, AalDV-3 (synonym: Aedes Peruvian densovirus APeDNV) (18), and HeDV (Hemagogus equinus densovirus HeDNV)—all associated with mosquito cell lines (19)—and another comprising AalDV-5, AalDV-2, AaeDV, CppDV, and AgDV, which were predominantly isolated from field mosquitoes (Fig. 2C) (20).

To determine whether the identified EMLs resulted from a laboratory-specific viral integration event occurring after cell line establishment, or instead represented authentic genomic integrations present prior to the derivation of the Aag2 cell line, we analyzed genomic DNA from Aag2 cells obtained from two independent laboratories in addition to our own. PCR amplification using primers targeting EML loci yielded identical products across all three sources, fully consistent with the publicly available Aag2 reference genome (Fig. 2D). These results strongly support that the MDV-derived EMLs are bona fide genomic integrations, likely present in the embryonic material from which the Aag2 cell line was derived, rather than arising during recent laboratory passage.

To examine whether Aag2-like MDV-derived EMLs are detectable in adult mosquitoes, we analyzed DNA prepared from the laboratory *Ae. aegypti* strain used in this study. Using EML-targeting primers, no EML-specific amplicons were detected in untreated adult mosquito samples, whereas the endogenous *AaeRps7* control was readily amplified. DNase-based nuclease treatment and ProK-plus-DNase treatment produced no EML-specific signal, while DNA from MDV-enriched Aag2 cells yielded the expected MDV amplicons. These results indicate that the tandem Aag2-like MDV-derived EMLs characterized in Aag2 cells were not detectable in the adult mosquito strain tested here (Fig. S1).

### The dynamics of EMLs

**Fig 3:**
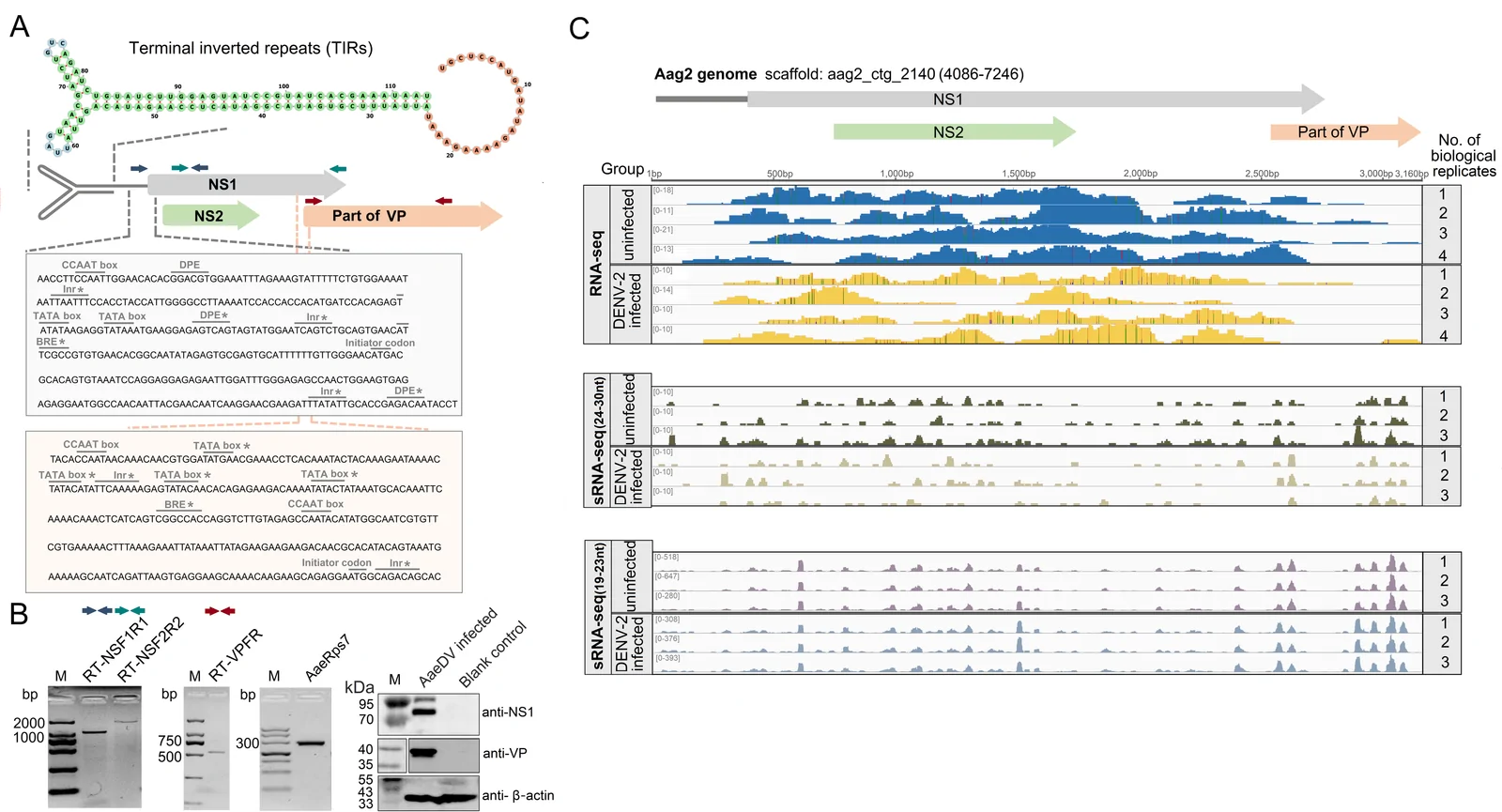
Transcriptional but not translational activity of EMLs in Aag2 cells. (**A**) Predicted cis-regulatory features of EMLs. The 5′-TIR adopts a canonical Y-shaped hairpin structure (predicted by RNAfold). Promoter analysis identified core elements (BRE, TATA, Inr, and DPE) upstream of NS1/NS2 and VP ORFs. RT-PCR detected three overlapping EML-derived transcripts spanning NS1 and partial VP from Aag2 cDNA. *AaeRps7* served as an intron-spanning control. (**B**) Western blot detection of MDV proteins. NS1 and VP proteins were readily detected in Aag2 cells acutely infected with AaeDV (positive control) but absent in naive Aag2 cells, confirming translational incompetence of EMLs. For the anti-VP blot, the molecular-weight marker lane and sample lanes were cropped from nonadjacent regions of the same membrane and are indicated by separate thin black boxes. The uncropped source blot is provided in the Uncropped Images supplemental file. (**C**) Small RNA-seq revealed limited 24–30 nt piRNA-sized reads overlapping EML loci, with low abundance and no obvious hotspot pattern. In contrast, 19–23 nt siRNA-sized reads were highly abundant but showed no infection-dependent changes in distribution or intensity.

To investigate whether the nearly full-length EMLs are transcriptionally active, we first searched for conserved cis-regulatory motifs within their sequences. As shown in Fig. 3A, the highly conserved 5′-terminal inverted repeat (5′-TIR) was predicted by RNAfold to fold into the canonical Y-shaped hairpin secondary structure, which in mosquito densoviruses is essential for genome replication, transcriptional regulation, packaging, and gene expression (21). Promoter analysis revealed that the regions upstream of the NS1/NS2 genes (pNS1 and pNS2, overlapping) and the VP gene (pVP) contained three core promoter elements—B recognition element (BRE), TATA box, and initiator (Inr)—as well as a downstream promoter element (DPE), further supporting their transcriptional competence.

We next assessed whether EMLs are indeed transcribed. Re-analysis of publicly available mRNA-seq data (SRR12200468–SRR12200475) showed that EML loci recruited over 100 uniquely mapping reads per sample in mock-infected groups, with pronounced coverage peaks spanning the NS and partial VP ORFs (Fig. 3C), indicating ongoing transcription (22). Notably, total read coverage decreased following DENV-2 infection. Experimental validation by RT-PCR further confirmed the presence of EML transcripts: three overlapping amplicons spanning nearly the full-length NS1 and partial VP regions were detected in Aag2 cDNA, while intron-spanning *AaeRps7* primers served as controls to exclude genomic DNA contamination (Fig. 3A).

EML loci exhibited limited piRNA-sized (24–30 nt) reads with low per-locus abundance and no evident hotspot pattern, suggesting weak or non-canonical piRNA production (23). In contrast, siRNA-sized reads (19–23 nt) were highly abundant but showed no infection-dependent changes in distribution or intensity (Fig. 3C).

Finally, we investigated whether EML transcription results in protein production. Because EMLs lack the 3′-TIR, the C-terminal portion of the VP ORF, and contain multiple premature stop codons in NS1, we hypothesized that protein translation would be impaired. Western blotting with antibodies against MDV NS1 and VP confirmed this: while Aag2 cells acutely infected with AaeDV displayed clear NS1- and VP-specific bands, naïve Aag2 cells showed no detectable signals (Fig. 3B). These findings indicate that although EMLs are actively transcribed, they are translationally defective, producing neither nonstructural nor capsid proteins.

### Effect of EML knockdown on arbovirus infection in Aag2 cells

**Fig 4:**
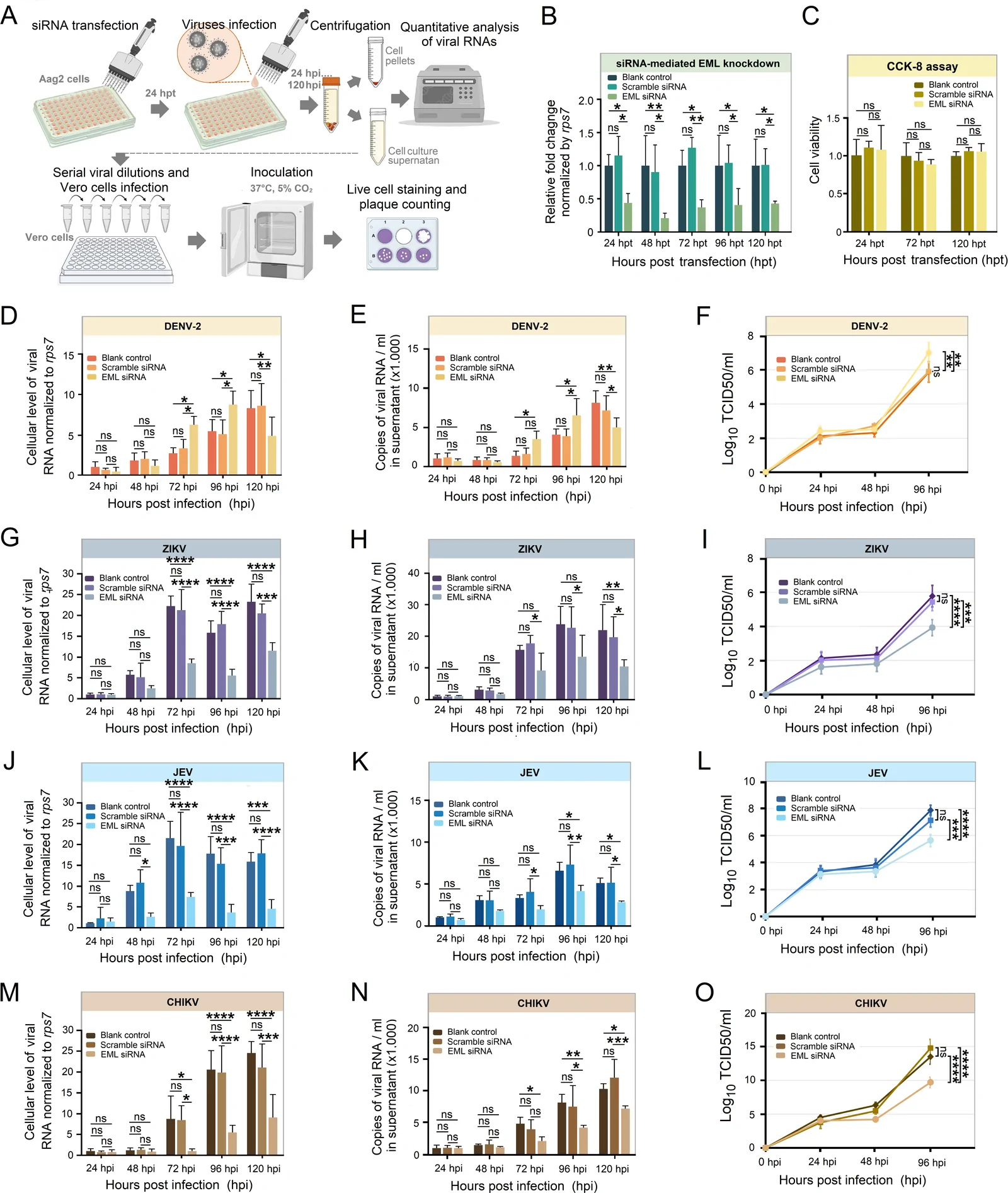
Effect of EML knockdown on arbovirus replication in Aag2 cells. (**A**) Experimental workflow. Aag2 cells were transfected with siRNAs for 24 h and then infected with DENV-2, ZIKV, JEV, or CHIKV (multiplicity of infection [MOI] = 1). Samples were collected between 24 and 120 h post-infection for qRT-PCR and TCID_50_ assays. (**B**) Knockdown efficiency of three siRNAs targeting EML transcripts, measured by RT-qPCR. (**C**) Cell viability analysis by CCK-8 assay showing no cytotoxicity of EML siRNA-2 compared with controls. (**D–F**) DENV-2: intracellular RNA, extracellular RNA, and TCID_50_ titers. (**G–I**) ZIKV: intracellular RNA, extracellular RNA, and TCID_50_ titers. (**J–L**) JEV: intracellular RNA, extracellular RNA, and TCID_50_ titers. (**M–O**) CHIKV: intracellular RNA, extracellular RNA, and TCID_50_ titers. Data represent mean ± SEM from three independent experiments. Statistical significance was determined by two-tailed Student’s *t*-test or one-way ANOVA (*P < 0.05; **P < 0.01; ***P < 0.001; ****P < 0.0001; ns, not significant).

Previous studies have shown that ISVs can modulate arbovirus replication and dissemination under co- or super-infection conditions through multiple mechanisms (24). To test whether EML transcripts exert similar effects, we designed three Cy3-labeled siRNAs (EML siRNA-1/-2/−3) targeting EMLs. Cy3 fluorescence confirmed cellular uptake of the three EML-targeting siRNAs (Fig. S2A), and RT-qPCR screening at 48 h showed that EML siRNA-2 produced the strongest knockdown (Fig. S2B). Subsequent time-course analysis showed that EML siRNA-2 reduced EML transcript levels by 75.3% at 48 h post-transfection, with knockdown declining to 30.1% by 120 h (Fig. 4B). Accordingly, EML siRNA-2 was used for subsequent assays. Cell viability assays (CCK-8) showed no significant cytotoxicity of EML siRNA-2 relative to negative-control siRNA or mock transfection at 24, 72, and 120 h (*P* > 0.05; Fig. 4C).

To determine the functional impact of EML silencing, Aag2 cells were transfected with EML siRNA-2 or control siRNA for 24 h and then infected (MOI = 1) with DENV-2, ZIKV, JEV, or CHIKV. Intracellular viral RNA was quantified by qRT-PCR, and infectious virus in the supernatant was measured by TCID_50_ between 24 and 120 h post-infection. For ZIKV, intracellular viral RNA levels were significantly reduced from 48 h onward, with parallel decreases in extracellular viral RNA and TCID_50_ titers becoming evident at 96 h (*P* < 0.05; Fig. 4G through I). For JEV, knockdown of EML transcripts led to a marked reduction in intracellular RNA beginning at 48 h, with significant declines in extracellular RNA at 48 h and TCID_50_ titers observed at 96 h (*P* < 0.01; Fig. 4J through L). For CHIKV, intracellular RNA reduction was detectable as early as 72 h, with consistent suppression of both intracellular and extracellular levels, including infectious titers, and reductions in TCID_50_ titers detected at 96 h (*P* < 0.05; Fig. 4M through O). In contrast, DENV-2 displayed a biphasic response: both intracellular and extracellular viral RNA were modestly elevated in EML siRNA-treated cells at 72 and 96 h (*P* < 0.05), but declined sharply by 120 h (*P* < 0.05; Fig. 4D and E). TCID_50_ measurements mirrored these patterns, showing transient enhancement followed by significant suppression at later stages (*P* < 0.05; Fig. 4D through F).

Collectively, these results demonstrate that EML transcripts facilitate efficient replication and release of ZIKV, JEV, and CHIKV while modulating DENV-2 replication in a temporally dynamic manner.

### Transcriptional changes following EML knockdown

**Fig 5:**
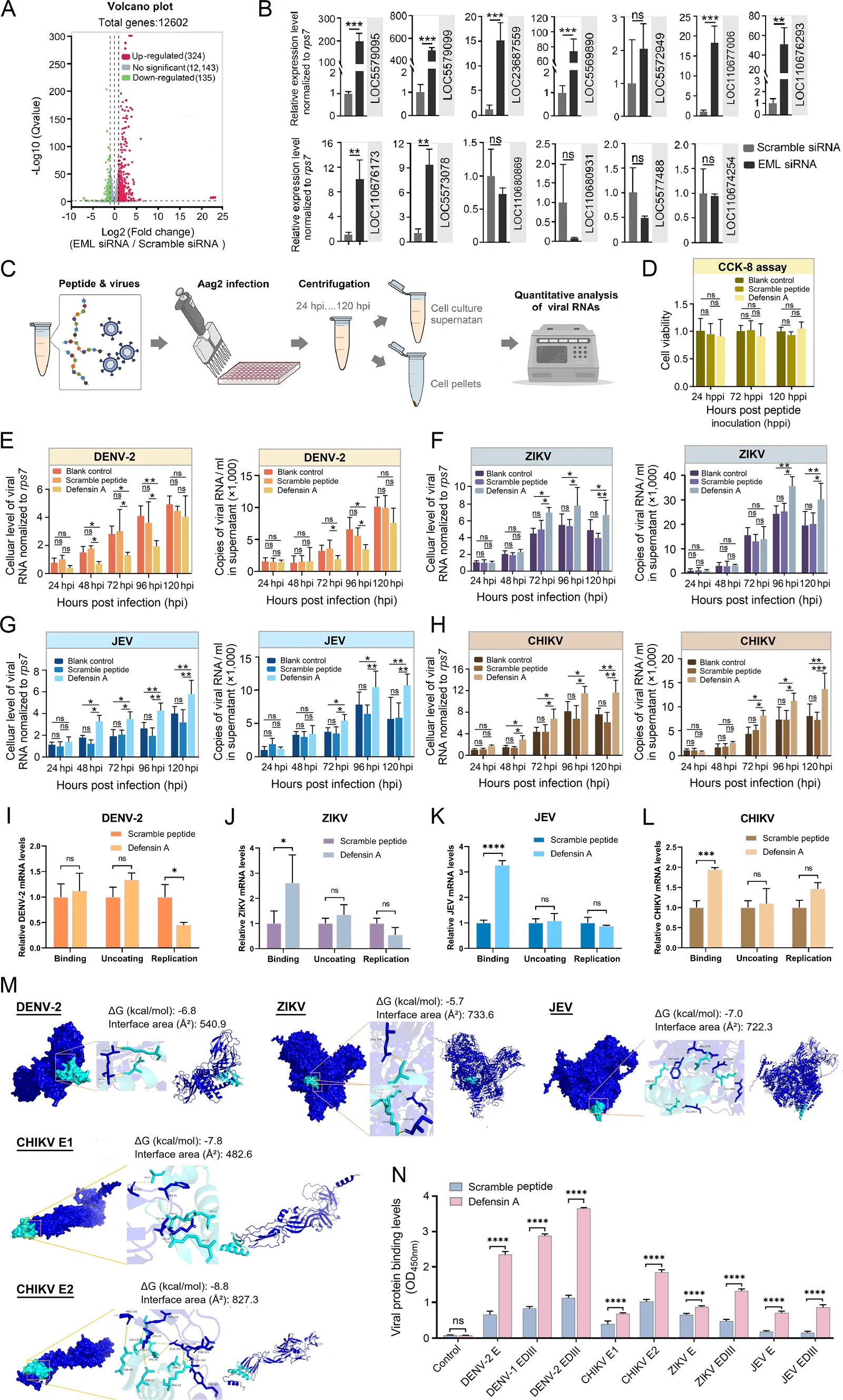
EML knockdown–induced transcriptomic profiling and synthetic defensin A validation identify defensin A as a stage- and virus-dependent modulator of arbovirus infection in Aag2 cells. (**A**) Volcano plot of DEGs following EML knockdown compared with negative control siRNA. (**B**) RT-qPCR validation of selected DEGs, highlighting strong upregulation of defensin-A-like (*LOC5579095*) and defensin-A (*LOC5579099*). (**C**) Experimental workflow for testing the effect of synthetic defensin A on arboviral RNA accumulation in Aag2 cells. (**D**) CCK-8 assay showing that treatment with synthetic defensin A did not affect Aag2 cell viability compared with scrambled peptide or blank control. Synthetic defensin A and scrambled peptide were used at 20 μM in panels C–L. (**E–H**) Effects of synthetic defensin A on arboviral RNA accumulation in Aag2 cells. For each virus, the left panel shows intracellular viral RNA levels normalized to *AaeRps7*, and the right panel shows extracellular viral RNA copies in the culture supernatant at the indicated time points: (**E**) DENV-2, (**F**) ZIKV, (**G**) JEV, and (**H**) CHIKV. (**I–L**) Stage-restricted defensin A treatment assays distinguishing adsorption-, entry-, and post-entry replication-stage effects for (**I**) DENV-2, (**J**) ZIKV, (**K**) JEV, and (**L**) CHIKV. Defensin A significantly enhanced adsorption-stage infection of ZIKV, JEV, and CHIKV, but did not significantly affect their entry- or replication-stage signals. In contrast, defensin A did not significantly affect DENV-2 adsorption or entry, but reduced DENV-2 RNA accumulation at the post-entry replication stage. (**M**) Molecular docking analysis predicting interactions between defensin A and the ED3 domains of DENV-2, ZIKV, and JEV, and the envelope glycoproteins E1 and E2 of CHIKV. Binding energies and interface areas are indicated. (**N**) Enzyme-linked immunosorbent assay (ELISA)-based binding assay showing that defensin A binds recombinant viral envelope proteins and receptor-binding regions more strongly than the scrambled peptide control across the tested arboviruses. Data are shown as mean ± SEM from three biological replicates. Statistical significance was assessed using Student’s *t*-test or one-way ANOVA (*P < 0.05; **P < 0.01; ***P < 0.001; ****P < 0.0001; ns, not significant).

To investigate whether EML depletion alters host transcription, we performed RNA-seq on Aag2 cells transfected with EML siRNA versus scrambled siRNA controls. Differential expression analysis identified 459 differentially expressed genes (DEGs; 324 upregulated, 135 downregulated; adjusted *P* < 0.05, |log_2_ fold change| ≥ 1) (Fig. 5A; Table S6). Gene Ontology analysis showed DEGs were enriched in cellular and metabolic processes, cell and organelle components, and functions like binding and catalytic activity. Kyoto Encyclopedia of Genes and Genomes (KEGG) pathway analysis indicated these genes were mainly involved in signal transduction, genetic information processing, metabolism, and human diseases, with significant enrichment in pathways such as cell cycle, MAPK, and PI3K-Akt signaling (Fig. S3).

For validation, 13 DEGs were selected using stringent criteria (*P* < 0.05, |log_2_ fold change| ≥ 3, and mean FPKM ≥ 100 for upregulated genes or |log_2_ fold change| ≥ 2 for downregulated genes) and analyzed by RT-qPCR. Among the upregulated genes were *LOC5579095* (defensin-A-like), *LOC5579099* (defensin-A), *LOC23687559*, *LOC5569890*, *LOC5572949*, *LOC110677006*, *LOC110676293*, *LOC110676173*, and *LOC557307*8. Notably, *LOC5579099* (*XM_001657243.3*) and *LOC110680759* (*XM_021856546.1*) share identical coding sequences; therefore, only *LOC5579099* is presented. Downregulated candidates included *LOC110680869*, *LOC110680931*, *LOC5577488*, and *LOC110674254*. RT-qPCR validation was generally consistent with the RNA-seq trends, with minor discrepancies in the magnitude of downregulation for *LOC110680931* and *LOC110674254*. Importantly, both *LOC5579095* and L*OC5579099* showed >100-fold induction in siRNA-treated cells compared to controls (Fig. 5B), highlighting defensin A as a prominent effector gene associated with EML knockdown.

Together, these results show that EML knockdown induces distinct transcriptional changes in Aag2 cells, most notably the strong upregulation of defensin A. This finding prompted us to investigate the functional role of defensin A in arbovirus infection.

### Defensin A differentially modulates arbovirus infection and directly binds viral envelope proteins

Given the strong upregulation of defensin-A-like (*LOC5579095*) and defensin-A (*LOC5579099*) upon EML knockdown, we next investigated the functional role of defensin A in arbovirus infection. Defensin A is a mature peptide of the insect defensin family (class 1 subfamily), consisting of 40 amino acids (ATCDLLSGFGVGDSACAAHCIARGNRGGYCNSKKVCVCRN) and characterized by six cysteine residues forming three disulfide bridges (25, 26). Although precursor protein sequences of *LOC5579095* (NP_001394965.1) and *LOC5579099* (NP_001394769.1) differ slightly, their mature peptides are identical. Thus, we synthesized the mature peptide to test both genes simultaneously. Synthetic defensin A and a scrambled control peptide (purity ≥99%) were used (Table S2).

We then examined the effect of synthetic defensin A on arboviral RNA accumulation in Aag2 cells (Fig. 5C through H). Aag2 cells were infected with DENV-2, ZIKV, JEV, or CHIKV at an MOI of 1. Experimental groups were treated with 20 µM defensin A, while negative and blank controls received scrambled peptide or PBS, respectively, with three biological replicates per condition. Viral genomic RNA was quantified in both cell pellets and supernatants by qRT-PCR. Intracellularly, ZIKV, JEV, and CHIKV showed significantly higher RNA copy numbers in defensin A–treated cells from 72 hpi onward (*P* < 0.05), whereas DENV-2 exhibited significantly lower intracellular RNA at 72 and 96 hpi (*P* < 0.05). Extracellular RNA levels mirrored these patterns: defensin A treatment increased ZIKV and CHIKV RNA at 72, 96, and 120 hpi (*P* < 0.05), elevated JEV RNA at 96 and 120 hpi (*P* < 0.05), but consistently reduced DENV-2 RNA at 72–120 hpi (*P* < 0.05). These results indicate that defensin A enhances ZIKV, JEV, and CHIKV infection but suppresses DENV-2 in a virus-specific manner.

To determine whether these effects were associated with a specific stage of infection, we separated peptide exposure into adsorption-, entry-, and post-entry replication-stage assays. When defensin A was restricted to the adsorption stage, subsequent viral RNA accumulation was significantly increased for ZIKV, JEV, and CHIKV compared with the scrambled peptide control, with the strongest effect observed for JEV, followed by CHIKV and ZIKV (Fig. 5J through L). In contrast, defensin A did not significantly alter the entry- or replication-stage signals of these three viruses. DENV-2 showed a distinct pattern: defensin A did not significantly affect DENV-2 adsorption- or entry-stage infection, but reduced DENV-2 RNA accumulation when added during the post-entry replication stage (Fig. 5I). These data indicate that the proviral effect of defensin A on ZIKV, JEV, and CHIKV is mainly associated with enhanced adsorption-stage infection, whereas its restrictive effect on DENV-2 occurs after entry.

A previous study reported that mosquito defensins directly bind the ED3 domain of the JEV envelope (E) protein, facilitating viral adsorption (27). To assess whether defensin A exhibits broader binding capacity across arboviruses, we performed molecular docking analyses using envelope proteins from representative flaviviruses and alphaviruses. For flaviviruses (DENV-2, ZIKV, and JEV), docking was conducted against the ED3 domain, a well-established receptor-binding region. In contrast, for the alphavirus CHIKV, docking was performed against the envelope glycoproteins E1 and E2, which together mediate viral attachment and membrane fusion. Docking predicted favorable interactions between defensin A and all tested viral envelope proteins (Fig. 5M), with contacts involving several surface-exposed residues, including THR-584/THR-585 and other residues indicated in the structural models. Quantitative docking analysis revealed binding energies (ΔG) of −5.7 to −8.8 kcal/mol and interface areas of 482.6–827.3 Å², suggesting that defensin A can engage envelope protein surfaces across distinct arbovirus families.

To experimentally validate these interactions, ELISA-based binding assays were performed using recombinant viral envelope proteins and receptor-binding domains. Defensin A exhibited significantly higher binding signals than scrambled peptide controls across all tested viruses (Fig. 5N), confirming its ability to directly associate with viral envelope proteins. Binding was detected with both full-length envelope proteins and receptor-binding regions, including flaviviral ED3 domains, supporting the ability of defensin A to target viral surface proteins.

Together, the binding and stage-restricted infection data indicate that defensin A–envelope association does not produce a uniform functional outcome across viruses. For ZIKV, JEV, and CHIKV, defensin A was associated with enhanced adsorption-stage infection. For DENV-2, however, strong envelope binding did not translate into increased adsorption or entry, suggesting a non-productive interaction with DENV-2 envelope surfaces. These results support a model in which defensin A acts as a non-canonical immune effector that binds viral envelope proteins but produces stage- and virus-dependent outcomes, rather than functioning solely through classical Toll or Imd signaling or as a uniformly proviral attachment factor.

### EML knockdown induces defensin A and remodeling of humoral immune gene expression

To investigate the mechanisms underlying the pronounced induction of defensin A following EML knockdown, we analyzed transcriptional changes in canonical antimicrobial peptide regulatory pathways and broader immune effector modules using RNA-seq data.

In insects, antimicrobial peptide expression is primarily controlled by the Toll and Imd signaling pathways, which activate NF-κB transcription factors (Rel1 and Rel2) to induce immune effectors including defensins, attacins, and gambicin (28–31). However, transcriptomic analysis indicated that EML knockdown did not produce a coordinated transcriptional activation of the canonical Toll or Imd pathways (Table S7). Several Toll-related receptors or ligands showed modest or mixed changes, whereas *MyD88* and *Rel1* remained largely unchanged. Interestingly, the NF-κB inhibitor *Cactus* was strongly upregulated, a pattern consistent with feedback regulation commonly observed in NF-κB signaling networks (32, 33). Similarly, genes associated with the Imd pathway showed only limited activation, with modest increases in *IKK2* and *Rel2* but minimal changes in *IMD*, *FADD*, and *PGRP-LC*.

Despite the limited transcriptional variation observed in these canonical signaling genes, defensin A and defensin A-like were among the most strongly induced genes following EML knockdown, showing more than two orders of magnitude increases relative to control cells. This response occurred together with coordinated upregulation of additional antimicrobial effectors, including defensin C, gambicin-like, attacin B, and several lysozyme-like genes, indicating activation of a broader antimicrobial effector program (Table S6).

Notably, EML knockdown also induced numerous genes associated with humoral immune amplification cascades, particularly CLIP-domain serine proteases and melanization-related proteases such as *CLIPB27*, *CLIPB21*, *CLIPB6*, *CLIPB22*, *CLIPB36*, *Hayan*, and *snake*, together with multiple proclotting enzyme–like genes (Table S6). In insects, CLIP serine protease cascades function as extracellular amplification modules that regulate immune processes including Spätzle activation, melanization, and systemic antimicrobial responses (34). Their coordinated induction therefore provides a plausible molecular framework for the strong upregulation of defensin A observed following EML silencing.

Consistent with these observations, several genes involved in immune recognition and humoral regulation—including *PGRP-LB*, *PGRP-S1*, and lectin-like genes such as *CTLGA5*—were also upregulated, suggesting broader remodeling of immune sensing and extracellular immune responses (Table S6). RT-qPCR validation further showed that *Cactus* expression increased significantly and *Rel2* showed a modest but significant increase, whereas the other tested Toll and Imd pathway components showed no significant transcriptional changes (Fig. S4).

Together, these findings indicate that the strong induction of defensin A following EML knockdown occurs without large transcriptional activation of canonical Toll or Imd pathway genes and is instead associated with coordinated enhancement of humoral immune amplification modules and antimicrobial effector programs.

### Simulation of EML-like transcripts suppresses defensin A expression and differentially alters arboviral RNA levels in C6/36 cells

**Fig 6:**
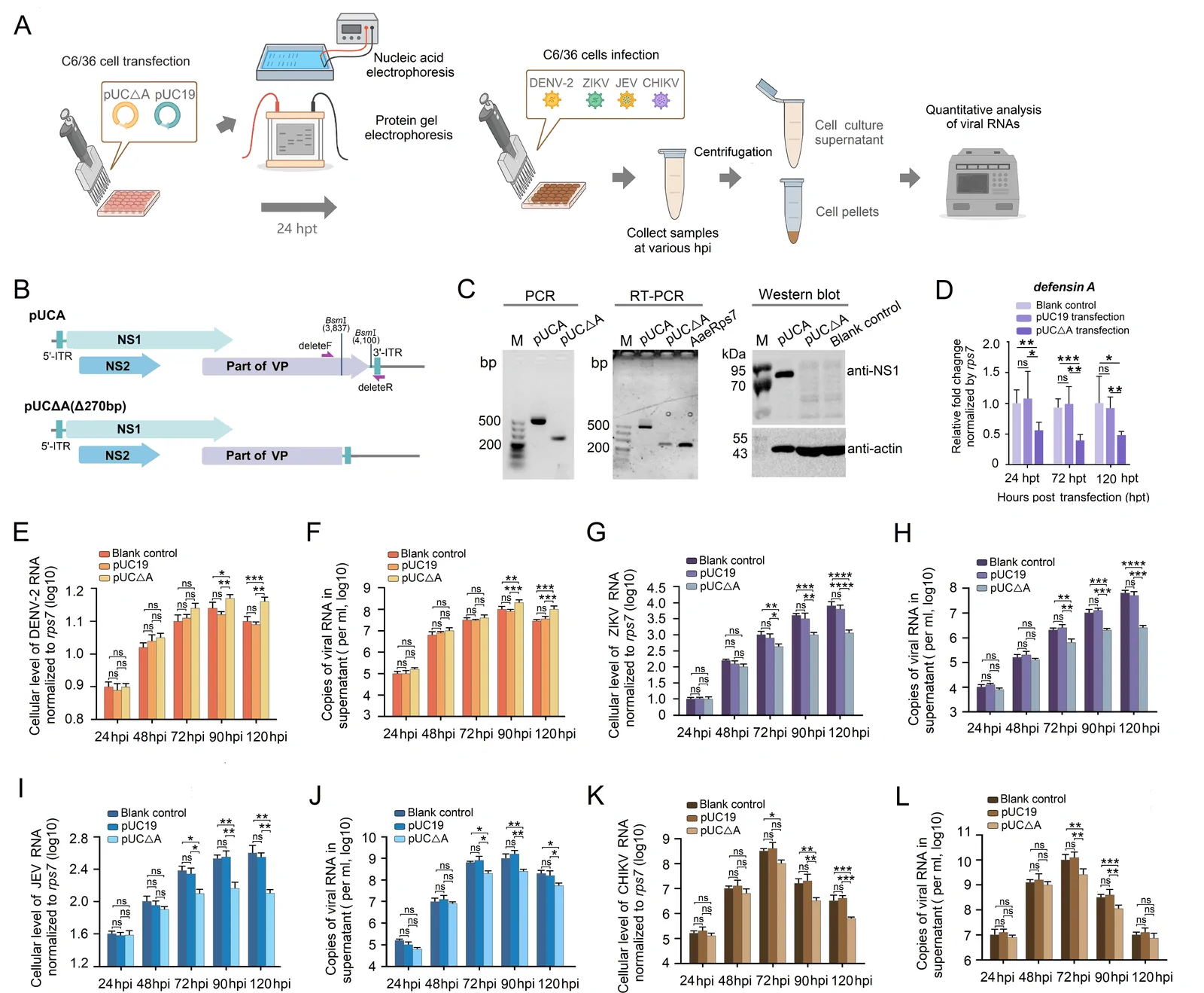
Simulation of EML-like transcripts suppresses defensin A expression and differentially alters arboviral RNA levels in C6/36 cells. (**A**) Workflow for simulating EMLs in C6/36 cells. Cells were transfected with the replication-deficient plasmid pUCΔA and subsequently infected with arboviruses. (**B**) Schematic of the pUCΔA construct, generated by deleting a 270 bp fragment within the VP gene from the infectious clone pUCA, yielding a transcriptionally active but translationally defective element. (**C**) Validation of pUCΔA. PCR confirmed the engineered deletion, RT-PCR verified transcript expression (primers spanning the deletion are indicated in panel B), and Western blot confirmed the absence of detectable NS1 protein. (**D**) Defensin A expression measured by RT-qPCR at 24, 72, and 120 h post-transfection, showing a significant reduction in the pUCΔA group compared with controls. (**E–L**) Intracellular (left panels) and extracellular (right panels) viral RNA levels in C6/36 cells transfected with pUCΔA, pUC19 (vector control), or left untreated, following infection with DENV-2 (**E and F**), ZIKV (**G and H**), JEV (**I and J**), or CHIKV (**K and L**). Data are presented as mean ± SEM of three independent experiments. Statistical significance was determined by Student’s *t*-test or one-way ANOVA (*P < 0.05; **P < 0.01; ***P < 0.001; ****P < 0.0001; ns, not significant).

To investigate the direct impact of EML expression on defensin A levels, we engineered a replication-deficient plasmid (pUCΔA) by deleting the partial 3′-end of the VP gene (270 bp) from the full-length Aedes aegypti densovirus clone pUCA. This design preserves viral transcriptional elements while disrupting productive viral protein expression and replication (Fig. 6B). Importantly, this construct was designed to model the functional state of endogenous EMLs rather than to reproduce their exact genomic structure. Specifically, pUCΔA represents a densovirus-derived sequence that remains transcriptionally active but is defective in producing functional viral proteins, thereby capturing the key biological feature of EMLs observed in Aag2 cells.

Because C6/36 cells lack the Aag2-like MDV-derived EMLs characterized here, they provide a useful heterologous system to assess the functional consequences of introducing an EML-like transcript. C6/36 cells transfected with pUCΔA expressed EML-like transcripts, as confirmed by RT-PCR using primers spanning the deleted region, but lacked detectable NS1 protein by Western blot, demonstrating successful simulation of a transcriptionally active but nonproductive viral element (Fig. 6C).

We next measured *defensin A* mRNA by qRT-PCR at 24, 72, and 120 h post-transfection. Relative to empty-vector controls (pUC19), defensin A expression in the pUCΔA group was significantly downregulated at all time points (Fig. 6D).

To further evaluate the functional role of simulated EML expression, C6/36 cells were transfected with pUCΔA, pUC19 (negative control), or left untreated (blank control), and infected with DENV-2, ZIKV, JEV, or CHIKV (MOI = 1). Viral genomic RNA was quantified in cell lysates and supernatants at 24–120 hpi (Fig. 6E through L). For DENV-2, pUCΔA transfection significantly increased intracellular RNA at 90–120 hpi (*P* < 0.05; Fig. 6E) and elevated extracellular RNA over the same interval (*P* < 0.01–0.001; Fig. 6F). In contrast, ZIKV RNA levels were markedly reduced, with intracellular RNA reduced from 72 hpi onward (*P* < 0.01; Fig. 6G) and parallel decreases in extracellular RNA at 72–120 hpi (*P* < 0.01–0.0001; Fig. 6H). For JEV, intracellular RNA was downregulated at 72–120 hpi (*P* < 0.05–0.01; Fig. 6I), accompanied by reduced extracellular RNA in the same period (*P* < 0.05; Fig. 6J). CHIKV also showed inhibition, with intracellular RNA reduced at 96–120 hpi (*P* < 0.05–0.001; Fig. 6K) and extracellular RNA decreased at 96–120 hpi (*P* < 0.01–0.001; Fig. 6L).

Collectively, these results show that introduction of a transcriptionally active but nonproductive densovirus-derived sequence is sufficient to suppress defensin A expression and alter arboviral RNA accumulation in a virus-dependent manner in C6/36 cells. Although this heterologous system does not fully reproduce the endogenous Aag2 EML context, it supports the principle that densovirus-derived RNA activity can modulate defensin-associated immune outputs independently of viral protein expression. Together with the Aag2 knockdown data, these findings link MDV-derived EML activity to defensin regulation and virus-specific infection outcomes.

## DISCUSSION

EVEs are widely distributed in mosquito genomes and have primarily been studied as sources of small RNAs contributing to antiviral defense (11, 35). Here, we uncover an additional and conceptually distinct function of EVEs: EMLs act as noncoding regulatory RNAs that modulate innate immune effector systems, thereby shaping arbovirus infection in a virus-dependent manner. These findings expand the functional scope of EVEs beyond piRNA-mediated immunity and position them as active regulators of host immune homeostasis.

Our data demonstrate that EMLs are transcriptionally active but translationally defective, consistent with their truncated genomic structure and lack of detectable viral protein production. Despite this, EML depletion triggered a strong transcriptional response, most prominently the dramatic upregulation of defensin A and defensin-A-like genes. This indicates that EMLs act through RNA-mediated mechanisms rather than protein-coding functions. Antimicrobial peptides such as defensins are increasingly recognized as context-dependent regulators of viral infection, capable of exerting either proviral or antiviral effects depending on the virus and cellular environment (36, 37). In mosquitoes, defensins have been implicated in JEV infection by promoting early virus-cell interactions, particularly viral adsorption (27), whereas DENV infection is strongly influenced by Toll/NF-κB-dependent immune restriction (31, 38). Consistent with this framework, our stage-restricted assays support a stage-specific model: defensin A promotes ZIKV, JEV, and CHIKV mainly at the adsorption stage, but restricts DENV-2 at a post-entry replication stage. Thus, the effect of defensin A is not determined by viral taxonomy alone, but by how defensin–virus binding is translated into productive attachment or downstream restriction.

These findings support a model in which EML transcripts function as immune “rheostats,” modulating the amplitude of humoral immune responses. Notably, EML knockdown did not strongly activate canonical Toll or Imd pathway components at the transcriptional level, as *Rel1*, *MyD88*, and *Pelle* remained largely unchanged, *Rel2* showed only a modest increase, and the NF-κB inhibitor *Cactus* was upregulated. This argues against a simple model of direct pathway activation. Instead, EML depletion induced a coordinated upregulation of antimicrobial effectors—including defensins, attacins, gambicin-like peptides, and lysozymes—together with multiple CLIP-domain serine proteases. These proteases are known to function as extracellular amplification cascades that link immune recognition to effector activation, including melanization and antimicrobial peptide production (34). Thus, EMLs likely regulate immune output at the level of network amplification and systemic immune tone, rather than through a single signaling pathway. Importantly, a replication-deficient densovirus-derived construct (pUCΔA) reproduced both defensin suppression and virus-specific effects in C6/36 cells. This construct was designed to model the functional state of EMLs—namely, a densovirus-derived sequence that remains transcriptionally active but is defective for productive viral protein expression and replication—rather than to reproduce all structural features of endogenous EMLs identified in Aag2 cells. Because pUCΔA was transcriptionally active but nonproductive, these data support that densovirus-derived RNA activity can modulate defensin-associated immune outputs independently of viral protein expression.

In addition to transcriptional regulation, our study provides evidence that defensin A directly interacts with arboviruses. Molecular docking predicted favorable interactions between defensin A and viral envelope proteins, including flaviviral ED3 domains and alphaviral envelope proteins, and ELISA-based assays supported direct association between defensin A and these viral proteins. The functional consequence of this binding, however, was virus- and stage-dependent. Stage-restricted assays showed that defensin A enhanced adsorption-stage infection of ZIKV, JEV, and CHIKV, without significantly affecting their entry or post-entry replication stages. In contrast, defensin A did not enhance DENV-2 adsorption or entry, but reduced DENV-2 RNA accumulation when applied after entry. These data indicate that defensin A–envelope interaction is not intrinsically proviral. For DENV-2, strong defensin A binding may represent a non-productive interaction that fails to support productive virion attachment and may instead be coupled to post-entry restriction.

The biphasic response of DENV-2 to EML knockdown—transient enhancement followed by later suppression—highlights the complexity of EML-mediated immune regulation. The stage-restricted assays indicate that defensin A does not enhance DENV-2 adsorption or entry, but instead reduces DENV-2 RNA accumulation at the post-entry replication stage. This finding provides a mechanistic basis for why DENV-2 differs from ZIKV, JEV, and CHIKV in response to defensin A. At the same time, the full EML knockdown phenotype cannot be attributed to defensin A alone. EML depletion induces broader humoral immune remodeling, including defensins, attacins, gambicin-like peptides, lysozyme-like genes, and CLIP-domain serine proteases. We therefore propose that the late suppression of DENV-2 reflects the combined effect of defensin A-associated post-entry restriction and broader immune amplification induced by EML depletion. This interpretation is consistent with the known sensitivity of DENV to Toll/NF-κB-linked immune responses and broader immune network interactions in mosquitoes (31, 38). Because our transcriptomic analysis was not time-resolved, the precise sequence of immune events underlying the early-to-late transition remains to be defined. Future time-course studies will be required to dissect the mechanisms underlying this transition.

Our findings have important implications for understanding mosquito–virus coevolution. Mosquito genomes contain abundant and dynamic EVEs derived from diverse viruses (11, 39). While these elements have primarily been linked to small RNA pathways, our results suggest that EVEs can also function as regulators of immune homeostasis. In this context, EMLs may represent a mechanism by which virus-derived sequences are repurposed to fine-tune immune responses, thereby influencing vector competence. Because vector competence is highly sensitive to immune regulatory thresholds, EVE-mediated modulation of immune tone could contribute to variation in susceptibility and transmission efficiency across mosquito populations. Together, these observations support the view that EVEs may be integrated into host immune regulatory networks rather than acting only as passive genomic remnants. They may therefore play a previously underappreciated role in shaping arbovirus transmission dynamics.

This study has several limitations. Although ELISA assays and stage-restricted infection assays support defensin–virus interaction and define the major stage affected by defensin A, additional biochemical and virological analyses are still required to determine whether defensin A alters receptor engagement, fusion, trafficking, or uncoating of DENV-2. In addition, the molecular pathway linking EML transcripts to immune regulation remains incompletely defined. Moreover, in the defensin A treatment, stage-restricted, and pUCΔA simulation experiments, viral outcomes were assessed primarily by viral RNA quantification, whereas infectious titers were not determined by TCID_50_. Therefore, these results should be interpreted as evidence of altered viral RNA accumulation rather than direct measurement of infectious virus production. Finally, our functional analyses were performed in mosquito cell lines. Although Aag2-like MDV-derived EMLs were not detectable in the adult *Ae. aegypti* strain tested here, this does not exclude the possibility that related MDV-derived elements may occur in other mosquito populations. Population-level genomic screening and *in vivo* functional studies will be required to determine whether related EMLs contribute to defensin regulation and vector competence in natural mosquitoes.

In summary, we identify MDV-derived EMLs as a previously unrecognized class of regulatory RNA elements that modulate mosquito innate immunity and differentially shape arbovirus infection through defensin-associated pathways. These findings extend the functional landscape of EVEs beyond small RNA-mediated immunity and reveal a new layer of host–virus interaction that links endogenous viral sequences to immune regulation, virus-specific infection outcomes, and potentially vector competence evolution.

## MATERIALS AND METHODS

### Cells and mosquitoes

*Aedes albopictus* C6/36 cells (ATCC, Manassas, VA, USA; Cat# CRL-1660, RRID: CVCL_Z230) were cultured at 28°C in RPMI-1640 (Gibco, NY, USA) supplemented with 10% fetal bovine serum (FBS; Gibco). *Aedes aegypti* Aag2 cells were maintained at 28°C in Schneider’s Drosophila medium with L-glutamine (Gibco) supplemented with 10% FBS (Cultilab). Vero cells (African green monkey, *Cercopithecus aethiops*, kidney fibroblast-derived; ATCC CCL-81) were grown in Dulbecco’s modified Eagle medium (DMEM) containing 5% (vol/vol) FBS at 37°C in 5% CO₂. Unless otherwise indicated, mosquito cells were passaged every 3–4 days at a 1:4 split; Vero cells at a 1:10.

*Ae. aegypti* (Haikou strain) were reared at 26°C ± 1°C, 75% ± 5% relative humidity, under a 14:10 light/dark photoperiod. Adults had continuous access to 10% sucrose. Females were blood-fed with defibrinated sheep blood (Solarbio Life Sciences, Beijing, China) using a Hemotek membrane feeder (Hemotek Ltd., Blackburn, UK).

### Viral stocks

The infectious clone of Aedes aegypti densovirus (AaeDV), pUCA (full-length AaeDV genome), was kindly provided by Prof. Erica Suchman and Jonathan Carlson; plasmid construction has been described (40). Wild-type AaeDV was rescued by transfecting pUCA into C6/36 cells as reported (40).

Arboviruses used included DENV-2 (New Guinea C strain; GenBank AF038403) (41); ZIKV (Rio-U1 strain; KU926309) (42); CHIKV (Ross strain; AF490259) (43, 44); and Japanese encephalitis virus (JEV, Nakayama genotype III; EF571853) (45). Working stocks were prepared by infecting near-confluent C6/36 monolayers (25 cm² flask) with virus diluted 1:6 in 3 mL RPMI-1640 containing 2% heat-inactivated FBS. Supernatants were harvested at ~3 days post-infection or upon cytopathic effect (CPE), clarified (1,500 × *g*, 5 min), aliquoted (0.5 mL), and stored at −80°C. Titers were determined as TCID_50_/mL by the Reed-Muench method (13).

### Biosafety and containment

All experiments involving infectious AaeDV, DENV-2, ZIKV, JEV, and CHIKV were conducted under biosafety level 2 (BSL-2) containment in accordance with institutional biosafety regulations. Virus propagation, infection assays, and collection of infected cell lysates or supernatants were performed by trained personnel in certified Class II biological safety cabinets using appropriate personal protective equipment. Work with infectious cultures was restricted to designated containment areas. Cultures, disposable materials, and liquid waste that contacted infectious virus were decontaminated by validated chemical disinfection and/or autoclaving before disposal. Samples were chemically lysed or otherwise decontaminated before removal from containment for downstream molecular analyses. Mosquitoes used in this study were maintained in contained insectary facilities with procedures to prevent escape, and no experiments involved infection of live mosquitoes with arboviruses. Recombinant viral envelope proteins used in ELISA were purified proteins and were handled as noninfectious material.

### Genome screening and validation of EMLs

The AaeDV reference sequence (GenBank M37899) was used to query the Aag2 genome assembly (GCA_021653915.1, VectorBase) using BLASTn. Hits with *E*-value <1 × 10^−6^ and length ≥250 nt were retained. Genomic DNA from Aag2 cells was extracted with the DNeasy PowerSoil Kit (QIAGEN, Hilden, Germany). Four primer pairs were designed to amplify overlapping fragments spanning predicted EML loci and flanking regions for validation by PCR and Sanger sequencing (Table S1A; Fig. 1).

### Detection of Aag2-like EMLs in adult mosquitoes

To examine whether Aag2-like MDV-derived EMLs were detectable in adult mosquitoes, DNA was prepared from adult *Ae. aegypti* mosquito homogenates from the laboratory strain used in this study. Untreated homogenate-derived DNA was analyzed together with DNase-treated samples and samples subjected to ProK treatment followed by DNase digestion. DNA from MDV-enriched Aag2 cells was included as a positive control. PCR was performed using two EML-targeting primer pairs located in the NS1 and VP regions, together with *AaeRps7* as an endogenous control. PCR products were analyzed by agarose gel electrophoresis.

### Molecular evolution analysis

NS1 protein sequences of the MDVs were aligned using Clustal X v1.81 (RRID: SCR_017055) (46); accession numbers are provided in Table S5. Phylogenies were inferred in MEGA11 (47) with the Neighbor-Joining method; branch support was assessed by bootstrap analysis (1,000 replicates) (48). Penaeus stylirostris penstyldensovirus 1 (PstDV-1) and Penaeus monodon hepandensovirus 1 (PmoHDV-1) were used as outgroups.

### Droplet digital PCR

Genomic DNA was prepared as described above. Copy numbers of EMLs were quantified on a QX200 Droplet Digital PCR System (Bio-Rad, Hercules, CA, USA) following the manufacturer’s protocols. Two single-copy *Ae. aegypti* reference genes, *AAEL002401* (49) and *AAEL006597* (50), were used for normalization. Primer sequences and cycling conditions are provided in Table S1A. Droplets were generated on the QX200 Droplet Generator, PCR was performed on a C1000 Touch Thermal Cycler (96-deep-well module), and droplet fluorescence was analyzed with QuantaSoft v1.7.4.0917 and QuantaSoft Analysis Pro v1.0.596. Template DNA was diluted as needed to fall within the linear dynamic range.

### RNA mapping and transcript validation

To evaluate the transcriptional activity of EMLs, multiple publicly available RNA sequencing data sets were analyzed. Bulk mRNA-seq data from Aag2 cells (NCBI SRA: SRR12200468–SRR12200475) were trimmed using Trimmomatic and aligned to EML reference sequences with STAR v2.7 under default parameters. Small RNA-seq data sets (SRR2633298, SRR3157917, and SRR2633591–SRR2633594) were further analyzed to determine whether EMLs give rise to small RNAs. Reads of 24–30 nt (piRNA-sized) and 19–23 nt (siRNA-sized) were mapped using Bowtie v1.2 (parameters: -v 1 -m 1). Enrichment of piRNA-sized reads overlapping EML loci was assessed, and genomic coordinates of mapped reads were annotated for downstream analysis.

For experimental validation, total RNA was extracted from Aag2 cells using TRIzol (Invitrogen, CA, USA), and cDNA was synthesized with PrimeScript RT Master Mix (Takara, Dalian, China). Three primer pairs—two targeting the NS1 region and one targeting the VP region—were used to detect EML-derived transcripts by RT-PCR and RT-qPCR. Amplification of the housekeeping gene *AaeRps7* was used as an internal control to exclude genomic DNA contamination and normalize expression levels.

### Western blotting

Polyclonal antibodies against MDV NS1 and VP were used as previously described (20). Total protein was extracted from Aag2 cells and from C6/36 cells transfected with pUCA (positive control). Proteins were separated by SDS-PAGE and transferred to PVDF membranes (Millipore, Bedford, MA, USA). Membranes were blocked in 5% (wt/vol) skim milk in PBST (PBS + 0.05% Tween-20) for 2 h at room temperature, incubated with primary antibodies (anti-NS1 or anti-VP, 1:1,000, overnight, 4°C), washed, and then incubated with HRP-conjugated goat anti-rabbit IgG (1:5,000; Santa Cruz Biotechnology, USA) for 2 h at room temperature. Signals were developed using Clarity Western ECL substrate (Bio-Rad, CA, USA). Western blot images were subjected only to linear adjustments applied uniformly to the entire image. When nonadjacent regions from the same membrane were cropped and juxtaposed for presentation, the marker and sample-lane regions were indicated by separate thin black boxes. In the anti-VP blot in Fig. 3B, the molecular-weight marker lane and sample lanes were run on the same gel and transferred to the same membrane in the same experiment but were nonadjacent on the original membrane. The uncropped source blot is provided in the Uncropped Images supplemental file.

### siRNA design, transfection, and RNA-seq

Three Cy3-labeled siRNAs targeting distinct EML regions were synthesized (Jieti Biotechnology, China). Aag2 cells were transfected with Lipofectamine 3000 (Invitrogen, Thermo Fisher Scientific, USA) per the manufacturer’s instructions; doses and time points are indicated in figure legends. Transfection efficiency (Cy3) and knockdown (RT-qPCR) were used to select the most effective siRNA. Sequences are listed in Table S1B.

For transcriptomics, total RNA from siRNA-treated and control cells was processed with the NEBNext Ultra RNA Library Prep Kit (NEB, USA) and sequenced at BGI Genomics (Shenzhen, China). Clean reads were aligned to the Aag2 reference genome with HISAT2; gene-level counts were obtained using the Subread package (51). Differential expression was analyzed with DESeq2 (52); genes with adjusted *P* < 0.05 and |log_2_(fold change)| ≥1 were considered differentially expressed.

### Acute arbovirus infection and TCID_50_ assay

Aag2 cells were seeded in 12-well plates 1 day before infection. After PBS wash, cells were inoculated with DENV-2, ZIKV, JEV, or CHIKV at an MOI of 1 as indicated, adsorbed for 15 min at room temperature and 2 h at 37°C, washed twice, and cultured at 28°C. Supernatant titers were determined by endpoint dilution on Vero cells (13). Briefly, Vero monolayers in 96-well plates (near-confluent) were inoculated with 10-fold serial dilutions of supernatants and incubated at 37°C, 5% CO₂. CPE was monitored daily, and TCID_50_/mL was calculated by the Reed-Muench method.

### Quantification of viral genome copy number

For AaeDV, samples were treated with TURBO DNase (Ambion, Austin, TX, USA) at 37°C for 1 h to remove non-encapsidated DNA, followed by extraction of encapsidated DNA using the Viral DNA Kit (Omega Bio-Tek, GA, USA). Genome copies were quantified by SYBR Green qPCR (SuperReal qPCR PreMix, Tiangen Biotech, Beijing, China) as described (14).

For arboviruses, total RNA was extracted with TRIzol, and cDNA was synthesized using the GoScript Reverse Transcription System (Promega, USA). Standard curves were generated from 10-fold serial dilutions of linearized plasmids containing virus-specific inserts. qPCR was performed with Maxima SYBR Green qPCR Master Mix (Thermo Scientific, USA) on a CFX96 system (Bio-Rad, USA). Primers used for quantification of DENV-2, ZIKV, JEV, and CHIKV are listed in Table S1A. All reactions were run in technical triplicate.

### Defensin A functional assay

Synthetic defensin A corresponding to the mature peptide sequence shared by defensin-A-like (*LOC5579095*) and defensin-A (*LOC5579099*), together with a scrambled control peptide, was synthesized and dissolved in sterile PBS. For functional assays, Aag2 cells were infected with DENV-2, ZIKV, JEV, or CHIKV at an MOI of 1 in the presence of 20 µM defensin A, scrambled peptide, or PBS. After inoculation, the inocula were replaced with Schneider’s medium containing 2% FBS supplemented with the corresponding peptide. At the indicated time points, intracellular viral RNA was extracted from cell pellets, and extracellular viral RNA was isolated from culture supernatants. Viral RNA levels were quantified by qPCR as described above.

### Stage-restricted defensin A treatment assays

To define the infection stage affected by defensin A, stage-restricted peptide treatment assays were performed in Aag2 cells. Cells were seeded in 24-well plates and used at approximately 90% confluence. DENV-2, ZIKV, JEV, and CHIKV were diluted 1:5 in Schneider’s medium containing 2% FBS. Synthetic defensin A or scrambled peptide was used at a final concentration of 20 µM, and each condition was tested with three biological replicates.

For the adsorption-stage assay, virus suspensions were preincubated with defensin A or scrambled peptide at 4°C for 2 h and then added to Aag2 cells for 4 h at 4°C. Cells were washed with pre-chilled PBS to remove unbound virus and cultured in fresh 2% FBS Schneider’s medium at 28°C for 48 h.

For the entry-stage assay, viruses were first adsorbed to Aag2 cells at 4°C for 4 h. After washing, cells were shifted to 28°C in medium containing defensin A or scrambled peptide for 6 h to allow entry/internalization. Cells were then washed again and cultured in fresh 2% FBS Schneider’s medium for an additional 42 h.

For the replication-stage assay, viruses were adsorbed to Aag2 cells at 4°C for 4 h, after which cells were washed and shifted to 28°C. Defensin A or scrambled peptide was added at 6 h after the temperature shift, and cells were maintained until 48 h after infection.

At the end of each assay, cells were lysed in TRIzol reagent. RNA extraction, cDNA synthesis, and virus-specific qPCR were performed as described above. Viral RNA levels were normalized to *AaeRps7* and compared between defensin A-treated and scrambled peptide-treated groups.

### Simulation of EML-like transcripts in C6/36 cells

The infectious clone pUCA (pUC19 carrying the full-length AaeDV genome, 3,981 nt) was kindly provided by Prof. Jonathan Carlson (53). To mimic endogenous MDV-derived EMLs, pUCΔA was generated by deleting a 270-bp fragment within the VP region of pUCA using BsmI digestion, end repair, and T4 ligation. The deletion was confirmed by Sanger sequencing and PCR with primers flanking the deleted region. C6/36 cells were transfected with pUCΔA, pUC19, or pUCA as indicated. pUCΔA-derived transcripts were detected by RT-PCR using primers spanning the deletion, and NS1 protein expression was assessed by Western blot, with pUCA serving as a protein-expression positive control. *Defensin A* mRNA levels were quantified by RT-qPCR at 24, 72, and 120 h post-transfection using *AaeRps7* as the reference gene. For arbovirus infection assays, C6/36 cells were transfected with pUCΔA or pUC19, or left untreated, and then infected with DENV-2, ZIKV, JEV, or CHIKV at an MOI of 1. Cell lysates and culture supernatants were collected at the indicated time points, and viral RNA levels were quantified as described above.

### Molecular docking analysis

Molecular docking was employed to explore potential interactions between defensin A and arboviral envelope proteins. For flaviviruses, amino acid sequences corresponding to the ED3 domains of DENV-2, ZIKV, and JEV envelope proteins were retrieved from UniProt. For CHIKV, the envelope glycoproteins E1 and E2 were used. Three-dimensional structures were predicted using AlphaFold 3 with default parameters. Protein–protein interaction predictions were performed using AlphaFold 3 in multimer mode. For each protein pair, five independent predictions were generated, and the top-ranked model was selected based on predicted alignment error (PAE) and interface confidence. PyMOL was used to visualize docking models, and interaction interfaces were analyzed using the PDBePISA server to estimate interface areas and predicted binding free energies. The predicted binding parameters are summarized in Table S4.

### ELISA-based viral protein–defensin A binding assay

To determine whether mosquito defensin A directly interacts with viral envelope proteins, ELISA was performed using purified recombinant viral proteins. High-binding 96-well ELISA plates were coated overnight at 4°C with synthetic mosquito defensin A peptide or a scrambled control peptide (5 µg/mL in 50 mM carbonate–bicarbonate buffer, pH 9.6). Plates were washed with PBST (PBS containing 0.05% Tween-20) and blocked with 2% BSA for 2 h at room temperature. Recombinant His-tagged viral envelope proteins and domains were kindly provided by Dr. Jianhai Yu from the Biosafety Level 3 (BSL-3) Laboratory, Southern Medical University (Guangzhou, China). The protein panel included E proteins and ED3 domains from DENV-2, ZIKV, and JEV, and E1 and E2 glycoproteins from CHIKV; all proteins were used at a final concentration of 2 µg/mL in blocking buffer.

After incubation for 2 h at room temperature, plates were extensively washed, and bound proteins were detected using mouse anti-His monoclonal antibody (1:1,000; ABclonal, Wuhan, China), followed by HRP-conjugated goat anti-mouse IgG (1:5,000; ABclonal). Color development was performed using TMB substrate (Lianke, Hangzhou, China), and the reaction was stopped with 2 M H₂SO₄. Absorbance was measured at 450 nm using a microplate reader. All experiments were performed with three independent biological replicates, each with technical triplicates.

### Statistical analysis

All experiments were performed in triplicate unless otherwise specified. Data are presented as mean ± standard error of the mean (SEM). Statistical analyses were conducted using two-tailed Student’s *t*-tests (two groups) or one-way ANOVA followed by post hoc tests (multiple groups) in IBM SPSS Statistics v23.0. A *P* < 0.05 was considered statistically significant: *P* < 0.05 (*), *P* < 0.01 (**), *P* < 0.001 (***), *P* < 0.0001 (****). Non-significant differences are indicated as ns. Distinct letters above bars denote significant differences (*P* < 0.05).

## Data Availability

Raw RNA-seq data generated in this study have been deposited in the NCBI Sequence Read Archive under BioProject accession number PRJNA1499400. Other data supporting the conclusions of this study are included within the article and its supplemental files.

## AFTER EPUB

*[This article was published on 11 September 2026 with errors in Acknowledgments and Funding. The errors were corrected in the current version, posted on 21 September 2026.]*

## SUPPLEMENTAL MATERIAL

Supplemental Figures msphere.00520-26-s0001.docx Figures S1 to S4.

Uncropped Images msphere.00520-26-s0002.docx Full uncropped western blot images supporting Fig. 3B and 6C.

Supplemental Tables msphere.00520-26-s0003.xlsx Tables S1 to S7.

ASM does not own the copyrights to Supplemental Material that may be linked to, or accessed through, an article. The authors have granted ASM a non-exclusive, world-wide license to publish the Supplemental Material files. Please contact the corresponding author directly for reuse.
