# Supplementary material for: MDV-like endogenous viral elements act as immune rheostats in Aedes cells by modulating defensin A-mediated responses to arboviruses
Source: mSphere. 2026 Sep 11;11(10):e00520-26. doi: 10.1128/msphere.00520-26 (PMC13711794; doi:10.1128/msphere.00520-26)
Supplement: Supplementary file 1 [file msphere.00520-26-s0001.docx]

**Supplementary Figure S1.** Detection of Aag2-like MDV-derived EMLs in adult *Ae. aegypti*. DNA was prepared from adult *Ae. aegypti* mosquito homogenates without treatment (mock), after DNase treatment, or after ProK treatment followed by DNase digestion. DNA from MDV-enriched Aag2 cells was included as a positive control. PCR was performed using two EML-targeting primer pairs located in the NS1 and VP regions, together with *AaeRps7* as an endogenous control. No EML-specific amplicons were detected in adult mosquito samples, whereas *AaeRps7* was amplified in untreated adult mosquito DNA. DNase-treated and ProK-plus-DNase-treated samples showed no detectable amplification. The positive control produced the expected MDV-specific bands.

**Supplementary Figure S2.** Efficiency of EML siRNA transfection and knockdown in Aag2 cells. (A) Representative images of Aag2 cells transfected with Cy3-labeled siRNAs targeting EMLs (EML siRNA-1, -2, -3) or a scrambled control siRNA at 24 h post-transfection. Bright field, Cy3 fluorescence, and merged channels are shown, indicating efficient siRNA uptake. Scale bar = 25 μm. (B) Knockdown efficiency of three EML-targeting siRNAs measured by RT-qPCR at 48 h post-transfection. Relative transcript levels were normalized to *AaeRps7*. EML siRNA-2 achieved the strongest silencing (81.8% reduction), followed by EML siRNA-1 (68.8% reduction) and EML siRNA-3 (not significant). Bars represent mean ± SEM from three independent experiments. Different letters indicate statistically significant differences. Bars sharing the same letter are not significantly different, whereas bars with different letters are significantly different (one-way ANOVA, *P* < 0.05).

**Supplementary Figure S3.** Functional enrichment analysis of differentially expressed genes (DEGs) following EML knockdown in Aag2 cells. (A) Gene Ontology (GO) classification of DEGs identified from RNA-seq analysis, categorized into biological process (BP), cellular component (CC), and molecular function (MF). (B) KEGG pathway enrichment analysis of DEGs, shown as a bubble plot comparing siRNA-treated (si) and control (nc) groups.

**Supplementary Figure S4.** RT-qPCR analysis of key Toll (*Cactus*, *Rel1*, *MyD88*, *Pelle*) and Imd (*Rel2*, *IKK2*, *IMD*, *PGRP-LC*) pathway components following EML knockdown in Aag2 cells. Expression levels were normalized to *AaeRps7* and shown relative to scrambled siRNA controls. EML knockdown significantly increased *Cactus* expression, while most canonical pathway components showed no significant transcriptional changes, with the exception of a modest increase in *Rel2*. Data represent mean ± SEM (n = 3). Statistical significance was determined by two-tailed Student’s t-test (**P* < 0.05; ****P* < 0.001; ns, not significant).
