# Supplementary material for: MDV-like endogenous viral elements act as immune rheostats in Aedes cells by modulating defensin A-mediated responses to arboviruses
Source: mSphere. 2026 Sep 11;11(10):e00520-26. doi: 10.1128/msphere.00520-26 (PMC13711794; doi:10.1128/msphere.00520-26)
Supplement: Supplementary file 2 [file msphere.00520-26-s0002.docx]

**Original Western Blots**

**Figure 3. Transcriptional but not translational activity of EMLs in Aag2 cells.**

**B**

**Original Western Blots**

**Figure 6. Simulation of EML-like transcripts suppresses defensin A expression and differentially alters arboviral RNA levels in C6/36 cells**

**C**
